# Carbon Monoxide Being Hydrogen Sulfide and Nitric Oxide Molecular Sibling, as Endogenous and Exogenous Modulator of Oxidative Stress and Antioxidative Mechanisms in the Digestive System

**DOI:** 10.1155/2020/5083876

**Published:** 2020-04-15

**Authors:** Edyta Korbut, Tomasz Brzozowski, Marcin Magierowski

**Affiliations:** Department of Physiology, Faculty of Medicine, Jagiellonian University Medical College, 16 Grzegorzecka Street, 31-531 Krakow, Poland

## Abstract

Oxidative stress reflects an imbalance between oxidants and antioxidants in favor of the oxidants capable of evoking tissue damage. Like hydrogen sulfide (H_2_S) and nitric oxide (NO), carbon monoxide (CO) is an endogenous gaseous mediator recently implicated in the physiology of the gastrointestinal (GI) tract. CO is produced in mammalian tissues as a byproduct of heme degradation catalyzed by the heme oxygenase (HO) enzymes. Among the three enzymatic isoforms, heme oxygenase-1 (HO-1) is induced under conditions of oxidative stress or tissue injury and plays a beneficial role in the mechanism of protection against inflammation, ischemia/reperfusion (I/R), and many other injuries. According to recently published data, increased endogenous CO production by inducible HO-1, its delivery by novel pharmacological CO-releasing agents, or even the direct inhalation of CO has been considered a promising alternative in future experimental and clinical therapies against various GI disorders. However, the exact mechanisms underlying behind these CO-mediated beneficial actions are not fully explained and experimental as well as clinical studies on the mechanism of CO-induced protection are awaited. For instance, in a variety of experimental models related to gastric mucosal damage, HO-1/CO pathway and CO-releasing agents seem to prevent gastric damage mainly by reduction of lipid peroxidation and/or increased level of enzymatic antioxidants, such as superoxide dismutase (SOD) or glutathione peroxidase (GPx). Many studies have also revealed that HO-1/CO can serve as a potential defensive pathway against oxidative stress observed in the liver and pancreas. Moreover, increased CO levels after treatment with CO donors have been reported to protect the gut against formation of acute GI lesions mainly by the regulation of reactive oxygen species (ROS) production and the antioxidative activity. In this review, we focused on the role of H_2_S and NO molecular sibling, CO/HO pathway, and therapeutic potential of CO-releasing pharmacological tools in the regulation of oxidative stress-induced damage within the GI tract with a special emphasis on the esophagus, stomach, and intestines and also two solid and important metabolic abdominal organs, the liver and pancreas.

## 1. Introductory Notes

### 1.1. Oxidative Stress

According to Sies et al., oxidative stress can be defined as an imbalance between oxidants and antioxidants in favor of the oxidants, leading to a disruption of reduction-oxidation (redox) signaling and control and/or molecular damage [1, 2]. Oxidative stress, in terms of its intensity, may be divided into eustress (physiological/positive stress) that plays several beneficial roles in physiological processes [1, 2] and excessive oxidative stress (distress/chronic/toxic stress) that may lead to the development and progression of pathological conditions [2–4]. Interestingly, recent reports indicated that oxidative stress can have a dual role in cancer development; on the one hand, it can promote molecular genetic alterations resulting in cancer cell transformation. On the other hand, it is also a necessary anticancer response-activating apoptotic pathway leading to selective cancer cell elimination [5, 6].

Reactive species, such as reactive oxygen species (ROS), nitrogen species (RNS), sulfur species (RSS), or carbonyl species (RCS), have a notable impact on redox signaling and oxidative stress [2, 4]. ROS, the most extensively studied component of oxidative stress, include free radicals such as superoxide radicals (O_2_^·−^) or hydroxyl radicals (OH^·^) and nonradical species such as hydrogen peroxide (H_2_O_2_) or singlet molecular oxygen (^1^O_2_) [2].

Under physiological conditions, production of ROS is highly restricted to specific enzymes that include the NADPH oxidases, xanthine oxidase, uncoupled endothelial nitric oxide synthase (eNOS), and the mitochondrial electron transport chain (mtETC) [7]. In order to protect themselves from ROS, the antioxidant defensive systems based on enzymatic or nonenzymatic components are activated and expressed within the cells. Enzymatic antioxidants, belonging to the first line of cellular defense system, include superoxide dismutase (SOD), catalase (CAT), and glutathione peroxidase (GPx). Nonenzymatic, the second line of defense, include glutathione (GSH) or vitamin E [3, 8]. However, it should be kept in mind that the abovementioned antioxidants may also contribute to oxidative damage. For instance, according to Sies et al., H_2_O_2_, an uncharged molecule, performs a major function in the course of oxidative stress rather than free radicals [9]. This is mainly due to its stability and ability to cross the cellular membranes and deliver a redox signal to distant targets [9]. Thus, enzymes such as SOD, catalyzing the conversion of O_2_^·−^ to H_2_O_2_, may have a dual effect: the first is a classical superoxide scavenger and the second can be the involvement of this enzyme in the regulation of ROS signaling [10].

Gastrointestinal (GI) tract is especially vulnerable to ROS attack due to constant contact with ingested materials and microbial pathogens. Moreover, ROS as well as RNS, such as nitric oxide (NO) and peroxynitrite (OONO^−^), are excessively produced during inflammatory states developed throughout the *digestive system* [11]. Oxidative stress has been implicated in a variety of major GI tract disorders including peptic ulcers; inflammatory bowel disease (IBD); and gastric, esophageal, and colorectal cancers [5, 12].

### 1.2. Carbon Monoxide

Carbon monoxide (CO) is a dangerous gas, produced by incomplete combustion of carbon-containing materials. It is tasteless, odorless, and colorless. CO binds to the hemoglobin (Hb) forming carboxyhemoglobin (COHb) with about 210 to 250 times greater affinity than that of oxygen. Such binding reduces the oxygen transportation ability of Hb leading to cellular hypoxia [13]. However, CO is also an endogenously generated gaseous mediator, which is produced during heme degradation *via* the activity of heme oxygenase (HO) enzymes. Currently, three main isoforms of HOs have been described, but only HO-1 and HO-2 have been defined as biologically active. HO-1 is a stress-inducible enzyme which represents a defense mechanism against oxidation and inflammation and is regulated by the transcription factor AP-1 activated by oxidative stress [14]. In contrast to HO-1, the isoform HO-2 is expressed constitutively [15]. Both, HO enzymes cleave the alpha-methylene carbon bond of the porphyrin ring of heme with the involvement of NADPH and molecular oxygen to yield equimolar amounts of biliverdin (BV), iron, and CO [16]. It is widely recognized that CO binds to a range of intracellular proteins containing heme prosthetic group, for instance, cytochrome c oxidase, cytochromes P450, myoglobin, guanylate cyclase, catalase, or cystathionine *β*-synthase (CBS) [16, 17].

It has been reported that endogenous CO-producing enzymes are expressed within the GI tract. Precisely, BV reductase (BVR) with the ability to convert BV to bilirubin and HO-2 are present in mucosal epithelial cells and in the endothelium of intramural vessels of the human gastric fundus [18]. Moreover, these proteins are localized in intramuscular interstitial Cajal cells (ICC) and in intrinsic nerve cell bodies of the submucosal and myenteric plexuses [18]. In rats, HO-2 was identified in gastrin cells of the pyloric glands and in parietal cells of the oxyntic glands [19]. Inducible HO-1 was shown to be expressed in mononuclear cells in the submucosa with minor staining in the epithelial cells of patients with ulcerative colitis [20]. HO-1 is expressed in endothelial cells of the mucous neck region of the gastric mucosa [21]. Moreover, this protein was localized in sinusoidal cells of the rat's liver [21]. Interestingly, pharmacological inhibition of HO by zinc protoporphyrin IX resulted in the attenuation of vasoactive intestinal polypeptide- (VIP-) induced low esophageal sphincter relaxation implying that this enzymatic protein contributes to the regulation of the motor activity of the upper GI tract [22–24].

**Figure 1:**
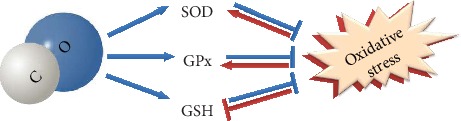
Schematic crosstalk between carbon monoxide and antioxidative enzymes. Arrows or blunt ends indicate activation or inhibition, respectively. Blue lines indicate CO-mediated processes; red lines indicate oxidative stress-mediated effects.

In the GI tract, the gaseous molecule CO has been shown to exert many physiological functions including its contribution to the mechanism of cell signaling, cytoprotection, regulation of microcirculation, motility, and modulatory effect of pathological events such as inflammation and carcinogenesis [25]. Moreover, exogenous and endogenous CO can be involved in redox signaling and initiate a compensatory expression of antioxidant enzymes and other adaptations to oxidative stress (Figure 1.) [15]. To summarize, the pleiotropic effect of CO, with an emphasis on redox biology, may improve clinical usefulness and applicability of CO-releasing molecules (CO donors) and their implementation in various therapeutic areas in the near future.

### 1.3. CO and Other Gaseous Mediators in Regulation of Oxidative Stress in the Digestive System

Endogenous CO, similarly to other two gaseous mediators, hydrogen sulfide (H_2_S) or nitric oxide (NO), can exert a variety of biologic and physiologic functions which range from the regulation of vascular tone, mitochondrial homeostasis and biogenesis, neurotransmission, the modulation of inflammation, programmed cell death to cellular proliferation programs [26]. However, CO, unlike NO and H_2_S, is not a free radical and does not alternate between different oxidative species; thus, it is considered more biologically stable [25, 27].

Interestingly, according to recently published data, the gaseous mediators CO, H_2_S, and NO were shown to play an important role within the GI tract [28, 29]. A large number of studies have focused on the contribution of these gaseous transmitters in the stomach's defensive response against gastric mucosal injury with special emphasis to possible interaction between them. For example, it has been reported that CO-releasing CORM-2, similarly to H_2_S released from NaHS, protected gastric mucosa against alendronate-induced damage in the gastric mucosa compromised by oxidation evoked by exposure to chronic mild stress [30]. Both mediators decreased the mRNA expression for nuclear factor *κ*B (NF-*κ*B) [30]; however, the direct interaction between the enzymatic pathways of endogenous H_2_S and CO still remained insufficiently explained. Interestingly, CO and H_2_S donors were demonstrated to reduce aspirin-induced gastric damage and lipid peroxidation observed as documented by an increase in the malondialdehyde (MDA) concentration in the gastric mucosa [31]. Similarly, NO was shown to attenuate nonsteroidal anti-inflammatory drug- (NSAID-) induced gastric bleeding [32, 33]. Pretreatment with NaHS and CORM-2 elevated gastric mucosal protein expression for antioxidative GPx but not for SOD [31]. Both molecules caused the antioxidative effects to be dependent on endogenous NO production [31]. Nevertheless, CORM-2-mediated gastroprotection and acceleration of ulcer healing was independent of H_2_S biosynthesis while NaHS was not effective when endogenous CO production was pharmacologically inhibited [34, 35]. It is worth to mention that CO and H_2_S donors were reported to protect the GI tract against acute oxidative damage induced by ischemia/reperfusion (I/R) injury [36, 37]. However, the NO/constitutive nitric oxide synthase (cNOS) pathway was shown to prevent I/R-induced gastric lesions while the activation of the NO/inducible nitric oxide synthase (iNOS) molecular pathway activity exacerbated this damage [38]. Interestingly, it was observed that H_2_S-releasing naproxen (ATB-346) exerted its GI safety as compared with the classic form of this drug in the gastric mucosa compromised by acute experimental stress due to modulation of gastric mucosal HO expression [39]. Taken together, all three gaseous mediators and their pharmacological donors afforded protective activity by the activation of antioxidative activity within the digestive system. However, the precise mechanism of possible interaction between these molecules in the context of oxidative stress modulation and prevention remains to be explained and requires further studies.

### 1.4. Carbon Monoxide Delivery Systems

There are various pharmacological and chemical tools available with the ability to modulate the concentration of CO *in vitro* and *in vivo*. This could include induction or inhibition of HO activity by hemin or zinc protoporphyrin IX, respectively [13]. However, recent approach has been concentrated on the pharmacological delivery of exogenous CO in a controllable manner and directly to the target tissue. It is worth to mention that the easiest way seems to be a systemic inhalation of a gas mixture containing CO but this concept is limited due to difficulties with storage and CO delivery in a controlled and directed manner [40–42]. Thus, Motterlini et al. proposed a series of transition metal carbonyls, termed CO-releasing molecules (CORMs) that are able to liberate CO and therefore to provide the direct biological effects to organs and tissues [41]. Being the first identified, the acronym CORM-1 (also known as DMDC) was assigned to dimanganese decacarbonyl (Mn_2_(CO)_10_). CORM-2 acronym was assigned to the tricarbonyldichlororuthenium (II) dimer ((Ru(CO)_3_Cl_2_)_2_) [42]. Both CORM-1 and CORM-2 are soluble in organic solvents. Moreover, CORM-2 and next-generation CORMs contain in their structure heavy metals, such as ruthenium that may potentially restrict their implementation into clinical pharmacology. In addition, the release of CO from these molecules requires photoactivation, as it is in the case of CORM-1 and ligand substitution for CORM-2 [42]. Therefore, novel water-soluble CO delivery molecules were described, tricarbonylchloro (glycinato) ruthenium II (RuCl(glycinato)(CO)_3_), termed CORM-3; boron-based compound Na_2_H_3_BCO_2_, named CORM-A1; or recently developed CORM-401 (Mn(CO)_4_ [43]) that in contrast to CORM-A1 releases up to three equivalents of CO per mol of the compound [42–44]. Additionally, the new class of organic CO-releasing prodrugs was developed recently [42, 45–47]. These CO prodrugs do not contain heavy metals, have a long half-life, and are able to release CO in a controllable manner. Importantly, few of them are activated to release CO only in contact with specific tissue enzymes, such as esterase and low pH on a click and release basis [42, 45–47]. Interestingly, some of these new compounds were developed as CO releasers in the presence of ROS [43].

## 2. CO and Oxidation within the Digestive System

### 2.1. Esophageal Mucosa

Esophageal mucosa is continuously exposed to external noxious agents and therefore is predisposed to epithelial damage [48]. Gastroesophageal reflux disease (GERD) resulting from the influx of the acidic stomach content into the esophagus is considered nowadays the global disease of the upper GI tract leading to the development of esophageal inflammation and oxidation [49]. It has been reported in rat models of reflux esophagitis that pretreatment with antioxidative isorhamnetin decreased esophageal lesion score reducing MDA levels, possibly due to the upregulation of the esophageal HO-1 expression [50]. Additionally, in cultured esophageal epithelial cells (EEC), it has been observed that euptailin prevented indomethacin-induced cytotoxicity and upregulated HO-1 expression due to nuclear translocation of transcription factor nuclear factor erythroid 2-related factor 2 (Nrf2) and the activation of extracellular signal-regulated kinases (ERKs) and phosphatidylinositol-3-kinase (PI3K)/Akt signaling [24, 51]. However, possible involvement of HO and CO and their possible interaction in the regulation of oxidative stress in the esophageal mucosa requires further investigations.

### 2.2. Gastric Mucosa

Stomach and gastric mucosa are important components of the GI tract, responsible for digestion, GI motility, and early microbial defense [52]. Oxidative stress, induced in response to exogenous gastric mucosal irritants, drugs, and pathogens derived from food intake, is one of the major contributors to the pathogenesis of gastric disorders such as gastritis, gastric ulcers, and gastric cancer and also drug-induced toxicity [53].

Importantly, the HO-1/CO pathway and CO donors has been considered one of protective factors involved in the protection of the gastric mucosa against numerous injuries mediated by oxidative stress. The most important antioxidative effects of CO donors in various *in vitro* and *in vivo* experimental models of gastric mucosa injuries were summarized in Table 1 with special attention paid to the dosages used and the form of pharmacological source of this gaseous molecule.

#### 2.2.1. HCl- and Ethanol-Induced Mucosal Damage

In an animal model of acute gastric mucosal lesions induced by the application of HCl, Ueda et al. have demonstrated that HO-1 mRNA expression level was upregulated and pretreatment with HO-1 inhibitor exacerbated the severity of these lesions [54]. Accordingly, Gomes et al. have evaluated the role of HO-1/BV/CO pathway in gastric mucosal defense against ethanol-induced gastric damage in mice [55]. They revealed the gastroprotective effects of hemin (HO-1 inducer), BV, and CO donor dimanganese decacarbonyl (DMDC) against the damage induced by this necrotizing agent by a mechanism involving a decrease in free radical production. Moreover, in mice treated with this CO donor, the reduced formation of MDA considered a marker of lipid peroxidation and increased GSH concentrations have been observed in the gastric mucosa with ethanol-induced gastropathy [55].

#### 2.2.2. Drug-Induced Mucosal Damage

Costa et al. have evaluated the gastroprotective effect of HO-1/CO pathway against alendronate-induced gastric damage in rats [56]. In their study, pretreatment with hemin or DMDC reversed the fall in gastric GSH levels and the rise in MDA level elevated after alendronate administration [56]. Thus, they concluded that CO may restore the mechanisms of redox balance and protects the gastric mucosa by the reduction in lipid peroxidation in this experimental model [56]. The question arises whether CO may play an important role in the protection of the gastric mucosa injured by the combination of ulcerogenic factors. Indeed, it has been demonstrated that CO released from CORM-2 is able to protect against alendronate-induced gastric lesions even when the gastric mucosa has previously been exposed to chronic mild stress [30]. In this chronic animal model, CORM-2 did not affect the mRNA expression of antioxidative enzymes GPx-1 and SOD-2 but decreased the expression of mRNA for oxidative marker NF-*κ*B, upregulated by treatment with alendronate in the gastric mucosa compromised by stress [30]. In another study [57], CORM-2 restored the activity of gastric mucosal antioxidant enzymes SOD and GSH, both decreased under stress conditions, and attenuated the expression of SOD-2 and GPx-1 mRNA, both markedly increased in the stressed gastric mucosa [57]. Additionally, pretreatment with CORM-2 exhibited beneficial effects in counteracting acute aspirin-induced gastric damage. CORM-2 inhibited gastric mucosal lipid peroxidation and restored antioxidative GPx-1 protein expression impaired by aspirin treatment, thus supporting an important role of CO in the protection of the gastric mucosa against oxidative injury [31]. Interestingly, in the same experimental model, CORM-2 abrogated the expression of proinflammatory cytokine IL-1*β* [31]. This pleiotropic cytokine IL-1*β* is associated with enhanced metastasis and poor prognosis of gastric cancer and was reported to stimulate the expression of IL-8, another inflammatory cytokine, through mitogen-activated protein (MAP) kinase and ROS signaling [31, 58]. Interestingly, hexacarbonyldicobalt derivative of aspirin considered a CO-releasing aspirin has been reported to decrease the ROS/RNS generation in malignant pleural mesothelioma (MPM) cell lines [59]. Taking into account the antioxidative activity of CO donors against NSAID-induced GI damage, we conclude that the development of novel safer CO-releasing derivatives of these drugs should be considered a new therapeutic option in limiting serious parent NSAID-induced complications which deserve attention of basic scientists and clinical practitioners.

#### 2.2.3. Gastric Cancer In Vitro Models

Besides *in vivo* animal models of ulcerogenesis, *in vitro* studies have been carried out to investigate the role of CO in gastric cancer. Lian et al. [60] have used CORM-2 (10, 25, and 50 *μ*M) to investigate the effect of CO on IL-1*β*-induced expression of IL-8 in human gastric cancer AGS cells. They observed that CORM-2 suppressed IL-1*β*-induced IL-8 expression and effectively inhibited IL-1*β*-induced ROS production determined by the H_2_O_2_-sensitive fluorophore DCFDA [60]. These observations support the notion that the antioxidant properties of CO and its ability to inhibit expression of proinflammatory cytokines such as IL-1*β*, can contribute, at least in part, to the gastroprotective effect of these gaseous molecules. However, it seems likely that the detailed mechanism by which CO attenuates ROS formation may strongly depend on the chosen experimental model and still remains to be elucidated.

### 2.3. Intestinal Mucosa

The intestine is responsible for digestion and absorption of nutrients, electrolytes, water, bile salts, and drugs. It also possesses immunological, endocrine, and motility response regulating functions [61]. The increased availability of CO levels in the intestinal compartment beneficially affects a course of various disorders, for example IBD, sepsis, postoperative ileus (POI), and outcomes following intestinal transplant in experimental animal models and preliminary studies in humans. Most of these diseases are directly or indirectly associated with inflammation and/or increased oxidative stress [62]. The most important antioxidative effects of CO donors in various *in vitro* and *in vivo* experimental models of intestinal mucosa injuries were summarized in Table 2.

#### 2.3.1. IBD

CO/HO-1 pathway and exogenous CO with its immunomodulatory properties and protective activities against oxidative stress reached increased importance due to its beneficial effects observed in the course of chronic intestinal inflammatory diseases, including the most common forms of IBD like ulcerative colitis (UC) and Crohn's disease (CD) [62–65]. For instance, the therapeutic potential of CO was evaluated by Takagi et al. in 2,4,6-trinitrobenzine sulfonic acid- (TNBS-) induced colitis in mice. They observed that increased colonic damage after TNBS administration was inhibited by the pretreatment with inhaled CO. Furthermore, CO significantly attenuated the production of thiobarbituric acid- (TBA-) reactive substances being interpreted in this study as an index of lipid peroxidation [66]. Additionally, Yin et al. examined the role of CORM-2 in a murine model of inflammatory colitis induced by the treatment with dextran sodium sulfate (DSS) [67]. Interestingly, to overcome drawbacks resulting from the poor aqueous solubility of CORM-2 and a very short CO-releasing half-life, a micelles consisting of water-soluble styrene-maleic acid copolymer (SMA) that encapsulated CORM-2 (SMA/CORM-2) were designed. SMA/CORM-2 polymers have shown significant therapeutic and tissue-protective effects, probably through CO released from the micelles evoking antioxidative and anti-inflammatory effects [67].

#### 2.3.2. Systemic Inflammation

Wang et al. have shown that exogenous CO can attenuate inflammatory responses in the small intestine of septic mice induced by cecal ligation and puncture [68]. Administration of CORM-2 significantly attenuated the production of proinflammatory cytokines (IL-1*β* and TNF-*α*) and suppressed lipid peroxidation in the small intestine of septic mice, considerably decreasing the formation of oxidants, and thus reducing the tissue oxidative injury. On the other hand, Liu et al. [69] employed CORM-2 to determine whether they can afford suppression of inflammatory cytokine production and oxidative stress in the small intestine of thermally injured mice. The application of CORM-2 on thermally injured mice decreased the production of IL-1*β*, TNF-*α*, and IL-8 and led to the significant downregulation of intestinal MDA tissue levels [69]. In addition, they also observed that GSH, a key antioxidant, declined significantly as compared to the control group, while treatment with CORM-2 reversed this effect [69]. Since the administration of CORM-2 prevents intestinal GSH depletion, it appears that the protective effect of CO donor involves the activation of an antioxidant defense system in protecting the intestinal tissue against oxidative stress [69]. Taken together, these studies seem to indicate that CORM-2 effectively prevents lipid peroxidation in the small intestine after experimental injury by decreasing the production of oxidants, which in consequence accounts for reduction of tissue oxidative injury.

#### 2.3.3. I/R Injury

The potent clinical CO-inducing protective effects have also been well documented in controlling intestinal I/R injury associated with transplantation. The GI organ damage caused by I/R is a significant problem in a variety of clinical settings usually associated with a high morbidity and mortality. Nakao et al. examined the efficacy of inhaled CO during intestinal cold I/R injury associated with small intestinal transplantation in rats. They observed that perioperative CO inhalation at a low concentration (250 ppm) resulted in the downregulation of several proinflammatory mediators and significantly increased antioxidant response in the intestinal graft, clearly indicating that in the CO-inhaled group less reactive oxygen metabolites were produced [70]. Similarly, Scott et al. suggested that a low dose of inhaled CO (250 ppm) may exhibit potent anti-inflammatory properties by inhibiting the production of proinflammatory cytokines [71]. They have also demonstrated a significant increase in ileum lipid peroxidation/oxidative stress following hindlimb I/R in male mice; however, as indicated by elevated MDA and remote intestinal mucosal injury, these events could not be efficiently prevented by a low dose of inhaled CO [71].

#### 2.3.4. POI

Transient impairment of gastrointestinal motility, termed POI, is a major determinant of recovery after abdominal surgery which leads to increased morbidity and prolonged patient hospitalization [72]. Backer et al. demonstrated that pretreatment with CORM-3 ameliorated the POI in surgically operated small intestine in mice. CORM-3 markedly reduced oxidative stress in both the intestinal mucosa and *muscularis propria*. Interestingly, pharmacological HO inhibition partially reversed the protective effects of CORM-3 on inflammation/oxidative stress in the *muscularis propria* and completely abrogated CORM-3-mediated inhibition of the early “oxidative burst” in the intestinal mucosa of POI. It has been suggested that this phenomenon might be related to the dysfunction of epithelial barrier and/or the different sources and amounts of ROS generation in the different layers of the intestine, for example, xanthine oxidase in epithelial cells of the mucosa versus NADPH oxidase in residential/infiltrated macrophages of the muscular layer [73]. To address these findings *in vitro*, studies by Babu et al. have proved the inhibitory influence of CO on ROS production in intestinal epithelial cells known to form a semipermeable barrier in the GI tract [74]. During inflammation, this barrier is at risk of damaging the effects of ROS, cytokines and microbial factors, and cytotoxins. In a mouse intestinal epithelial cell line MODE-Κ, TNF-*α*/cycloheximide (CHX) was used to induce oxidative stress as manifested by the increased ROS production and the decreased cellular levels of GSH [74]. These effects were partially prevented by treatment with CORM-A1 and correlated with diminished apoptosis and cell death, suggesting that modulation of ROS/oxidative stress might be considered a primary mode of action responsible for the antiapoptotic and cytoprotective effects of CO [74, 75]. Moreover, CORM-A1 acted solely on NADPH oxidase-derived ROS without major influence on the mtETC [76]. Nevertheless, the chemical characteristics of different CORMs have a nonnegligible effect on cellular regulation of ROS sources. As an example, Babu et al. revealed that the cytoprotective effect of water-soluble CORM-401 mitigates NADPH oxidase-derived ROS, whereas lipid-soluble CORM-2 interferes with both NADPH oxidase- and mitochondria-derived ROS to protect MODE-K cells from TNF-*α*/CHX-induced cell death [44, 76].

#### 2.3.5. Colon Cancer In Vitro Models

It is widely known that ROS induces DNA damages and different genetic disorders that are critical causes of cancers including colorectal cancer [77]. Dijkstra et al. have observed that in the human colon carcinoma DLD-1 cell line, HO-1 is strongly activated by various oxidative stress-inducing factors, including thiol-modifying agent diethylmaleate (DEM) and the lipid peroxidation end product: 4-hydroxy-nonenal (4-HNE) [14]. Interestingly, they have demonstrated a switch from a NF-*κ*B-regulated to an activator protein 1- (AP-1-) regulated stress response, which may be controlled by HO-1-derived CO [14].

### 2.4. Liver

The liver plays a crucial role in all metabolic processes and detoxifies endogenous compounds and xenobiotics, as a part of the digestive system. Therefore, this organ remains at the high risk of oxidative injury caused by the production of ROS. Oxidative stress has been considered a key factor causing liver damage induced by a variety of chemical and nonchemical agents, including alcohol, drugs, hepatic viral infections, and nutritional components, which in turn causes progression of hepatic injury, liver fibrosis, cirrhosis, and in some cases hepatocellular carcinoma [78–81]. These highly reactive species can be responsible for hepatic I/R injury occurring during surgical procedures such as liver resection and liver transplantation [82, 83]. The most important antioxidative effects of CO donors in various *in vivo* and *in vitro* experimental models of liver injury were summarized in Table 3.

#### 2.4.1. I/R Injury

Recently, the HO-1/CO system has been investigated as a potential mechanism for protection against oxidative stress and hepatic injury in numerous experimental models [84]. Brugger et al. provided evidence that CO inhalation (250 ppm) or administration of methylene chloride (MC) can reduce hepatic lipid peroxidation, reestablish total hepatic glutathione and glutathione disulfide (GSH/GSSG) ratio, and reduce hepatocellular injury in a murine model of bilateral hindlimb I/R [85]. The inhalation of CO during the reperfusion period and the oral gavage of MC caused a significant increase in COHb content. Moreover, these authors have attributed the observed reduction in hepatic ROS formation following CO administration to the inhibition of NADPH oxidase caused by this gaseous molecule [85].

Beneficial effects of CO were also observed by Lee and colleagues [86] in liver grafts initiated by cold preservation and augmented by reperfusion. They have shown using an *in vitro* model that exposure to 20% CO-containing medium for 6 h inhibited ROS generation in Kupffer cells (KC) under hypothermic condition with an upregulation of HSP70 protein [86]. Moreover, pretreatment with inhaled CO (250 ppm, for 24 h before liver graft retrieval) upregulated hepatic HSP70 protein expression and caused significant inhibition of cold I/R injury after liver transplantation *in vivo* [86]. It was demonstrated that CO bound to red blood cells (CO-RBC) exhibited the potential to protect hepatic cytochrome P450 protein, maintaining its ability to exert resuscitative effect in a rat model of hemorrhagic shock. This beneficial effect was attributed to the inactivation of KC resulting in the suppression of ROS production [87]. On the other hand, Kato have shown that exogenous supplementation with a low dose of bilirubin, an antioxidant bile pigment, rather than CO could be a crucial factor that significantly reduces oxidative stress and ameliorates I/R-induced hepatobiliary dysfunction in rats [88].

The signaling pathway by which CO can protect liver tissues against I/R-injury was studied by Kim et al. [89]. It has been reported that inhaled CO (250 ppm) attenuated liver damage *via* ROS-dependent Akt signaling and by the inhibition of glycogen synthase kinase 3*β* (GSK-3*β*) through Ser9 phosphorylation in the murine model of hepatic warm I/R-induced injury [89]. Moreover, CO ameliorated hepatic I/R injury by the regulation of miR-34a/Sirtin1 pathway known to modulate inflammation and apoptosis in response to oxidative stress [90]. These data strongly support the conclusion that increased bioavailability of CO by treatment with CO donors could be the promising preventive strategy against I/R injury after liver transplantation and may provide novel clinical opportunity in the management of liver disorders due to CO exerting antioxidative and anti-inflammatory properties.

#### 2.4.2. Alcoholic and Nonalcoholic Liver Damage

Besides important protective role against I/R injury, CO also conferred substantial prevention against alcoholic liver damage [91]. In adult male Balb/c mice treated with ethanol or incubated with ethanol primary rat hepatocytes, CO derived from HO-1 or released from CORM-2 exerted a substantial antioxidant action against oxidative damage in these experimental models of hepatic injury. This CO-induced protection was mainly manifested by suppressed lipid peroxidation, normalized GSH concentration, and SOD activity [91]. Furthermore, Upadhyay et al. investigated the therapeutic potential of CORM-A1 in acetaminophen- (APAP-) induced liver injury in mice [79]. They showed elevated levels of serum transaminases, depleted hepatic GSH, and hepatocyte necrosis after APAP treatment [79]. On the contrary, in mice injected with CORM-A1 after APAP administration, the reduction in serum transaminases, preservation of hepatic GSH, and attenuation of hepatocyte necrosis have been observed. Interestingly, mice that received a lethal dose of APAP died but those cotreated with CORM-A1 showed a 50% survival [79]. Additionally, CORM-A1 prevented hepatic steatosis in high-fat high-fructose (HFHF) diet fed mice, used as a model of nonalcoholic steatohepatitis (NASH) [92]. The beneficial effects of CORM-A1 in HFHF fed mice were associated with improved lipid homeostasis, Nrf2 activation, upregulation of antioxidant-responsive (ARE) genes, and increased ATP production [92].

The effects of HO-1 and its enzymatic activity products CO, BV, and iron/ferritin were also assessed in a mouse model of inflammatory liver damage induced by bacterial wall cytotoxin lipopolysaccharide (LPS) and hepatocyte-specific transcription inhibitor D-galactosamine (GalN). It has been shown that oral administration of the MC or BV was effective in the protection of hepatic damage in mice, prolonged their survival, and reduced the expression of proinflammatory cytokines (TNF-*α*, IFN-*γ*) [93]. Moreover, when GalN/LPS were administered to induce acute liver damage, the intraperitoneal injection of exogenous CO gas improved the survival rate of mice and attenuated hepatocellular damage. Exogenous CO administration markedly reduced MDA concentrations and restored SOD and GSH levels, thus inhibiting lipid peroxidation, which might considerably contribute to the mechanism of CO-mediated hepatoprotection [94].

#### 2.4.3. Hepatocyte In Vitro Models

The important role of CO in the maintenance of hepatic function in both physiological and pathophysiological conditions was also demonstrated in multiple *in vitro* studies. For instance, Lee et al. have suggested that CO induces Nrf2 activation *via* MAP kinase signaling pathways, thereby prompting an increase in HO-1 expression in HepG2 cells [95]. Similarly, Upadhyay et al. have revealed that CORM-A1 (10-100 *μ*M) facilitated nuclear translocation of Nrf2, reduced oxidative stress, u-regulated ARE genes, and prevented GSH depletion promoting cell viability in HepG2 cells treated with tert-butyl hydroperoxide (t-BHP), known to cause oxidative stress-mediated hepatocyte injury [79]. Moreover, Kim et al. [96] have determined whether the effects of CO are dependent on modulation of ROS signaling in primary rat- or mouse-derived hepatocytes and Hep3B cells. They found that CO treatment (250 ppm) triggered a low level of ROS production in hepatocytes *in vitro*, considered an adaptive response leading to an increase in cell viability, in combination with Akt phosphorylation and I*κ*B degradation (required for NF-*κ*B activation) [96]. This finding generated in cultured hepatocytes indicates the existence of another survival pathway, possibly parallel to Nrf2 activation [97]. Moreover, exogenous CO failed to increase ROS production in respiration-deficient Hep3B cells, suggesting that the mitochondria are the source of CO-induced ROS generation in this model [96].

#### 2.4.4. Liver Mitochondria

Recently, the possible contribution of mitochondria as the molecular targets of CO has been suggested [92, 96, 98]. These key organelles for cell energy supply play a crucial role in the initiation and progression of many diseases following oxidative stress-induced damage [98]. Piantadosi et al. revealed that exposure to gaseous CO (50 ppm) for 1, 3, or 7 days induced hypoxia-sensitive protein expression for hypoxia-inducible factor 1*α* (HIF-1*α*), HO-1, and SOD-2 in rat liver mitochondria [98]. CO was shown to induce a profound early mitochondrial oxidative stress manifested by a decrease in GSH/GSSG ratio and the activation of the mitochondrial pore transition (MPT) [98]. On the other hand, Queiroga et al. have demonstrated that low concentrations of CO (10 *μ*M) may inhibit mitochondrial membrane permeabilization (MMP) in isolated mouse liver mitochondria *in vitro*, possibly by preventing mitochondrial swelling, mitochondrial depolarization, and the opening of a nonspecific pore through inner membrane [99]. In addition, CO increased mitochondrial ROS generation that is essential for signaling of MMP inhibition, although not enough to induce the damage [99]. Moreover, CORM-A1 significantly ameliorated mitochondrial function in palmitic acid- (PA-) treated HepG2 cells *via* Nrf2 translocation and activation of cytoprotective gene expression. Furthermore, in PA-treated cells, CORM-A1 improved mitochondrial oxidative stress, mitochondrial membrane potential, and rescued mitochondrial biogenesis [92].

The abovementioned findings appear to indicate that CO may have a dual role in oxidative stress and its pro- or antioxidant effects depend on the dosage, route of administration, the exposure duration, and cell type.

### 2.5. Pancreas

The pancreas is an important organ for proper nutrient metabolism that consists of exocrine cells producing digestive enzymes and endocrine cells responsible for generation of pancreatic hormones. Malfunction of the exocrine part can lead to the development of pancreatitis and even pancreatic cancer [100, 101]. The most important antioxidative effects of CO donors in various *in vivo* and *in vitro* experimental models of pancreatic injury were summarized in Table 4.

#### 2.5.1. Acute Pancreatitis

Sato et al. have analyzed protein expression of the heme oxygenase in a rat model of acute pancreatitis showing that the expression of HO-1 in the pancreas *in vivo* was enhanced. Oxidative stress also elevated HO-1 expression level in murine islet (LTC3) and rat acinar (AR42J) pancreatic cells. These findings indicate that HO-1 may act as a potential inflammatory biomarker and a crucial defense mechanism against oxidative stress in acute pancreatitis [102]. It is noteworthy that ROS are possible regulators of pancreatic injury development. They may activate NF-*κ*B that regulates gene expression of numerous inflammatory markers [40, 101]. Chen et al. have demonstrated that CORM-2-releasing CO exerts beneficial effects on severe acute pancreatitis in rats. Of note, CORM-2 not only reduced the serum levels of proinflammatory TNF-*α* and IL-1*β* but also suppressed pancreatic tissue mRNA expression of TNF-*α* and IL-1*β*, whereas anti-inflammatory cytokine IL-10 was considerably increased. Interestingly, CORM-2 was also found to suppress NF-*κ*B binding activity which might testify for the protective, anti-inflammatory, and antioxidative effects of CO in this experimental model [103]. Similarly, Nuhn et al. have demonstrated that treatment with HO-1 metabolites has a beneficial influence on severity and survival of acute necrotizing pancreatitis in rats induced by retrograde intraductal injection of sodium taurocholate [104]. Biliverdin hydrochloride (BV-HCl), the CO donor MC, or iron-chelating desferrioxamine (DFO) was used in this model [104]. All HO-1 metabolites showed protective effects on the severity of pancreatitis accompanied by the diminished pancreatic NF-*κ*B activity [104]. In turn, Nagao et al. have examined the therapeutic efficacy of CO-bound Hb vesicle (CO-HbV), a CO carrier, against severe acute pancreatitis in mice that were fed with a choline-deficient ethionine-supplemented diet. A CO-HbV treatment significantly reduced mice mortality with experimental acute pancreatitis by inhibiting the systemic release of proinflammatory cytokines, neutrophil infiltration, and locally oxidative injuries to the pancreatic tissue [105]. Therefore, the administration of HO-1 products, including CO, seems to decrease oxidative stress and attenuate the inflammatory changes in acute pancreatitis.

#### 2.5.2. Autoimmune Diseases

Interestingly, CO was also identified as a potential therapeutic molecule for the treatment of diseases related to pancreas autoimmune diseases, such as type 1 diabetes. Recently, Nicolic et al. have shown that CORM-A1 suppressed the incidence and the severity of immunoinflammatory and autoimmune diabetes in experimental mouse models of type 1 diabetes [106]. Moreover, Li et al. revealed that the upregulation of HO-1 decreased superoxide (O_2_^· −^) generation and increased CO release and bilirubin formation in the pancreas of nonobese diabetic mice [107]. Taken together, these results indicate that enhanced HO-1 activity associated with increased production of CO can significantly counteract the diabetic complications [107].

## 3. Summary

**Figure 2:**
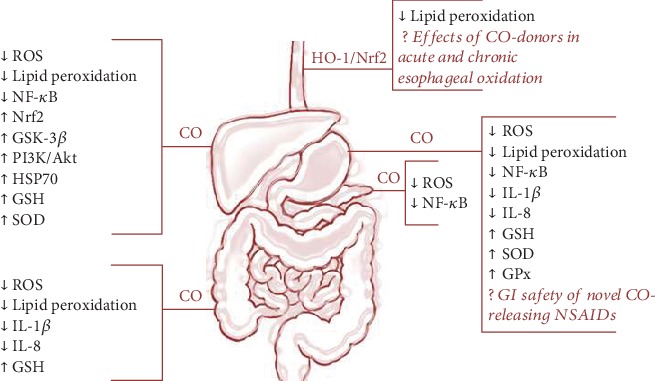
Pleiotropic effects of HO-1/CO pathway against oxidative stress in the digestive system.

As presented in this review, the gaseous molecule CO plays an essential physiological role exhibiting beneficial pleiotropic effects in the maintenance of GI tract integrity and the mechanism of GI mucosal defense (Figure 2). Bioavailability of CO released from its donors seems to depend on many factors, such as dosage of this CO donor, the exposure time, and mechanism of its release by particular donors. Different CO sources may give rise to distinct and complex responses in different parts of the digestive system.

Nevertheless, according to an evidence-based medicine, the major mechanism of the beneficial action of this gaseous molecule depends upon cell oxidative metabolism, modulation of ROS generation, and antioxidative activity as reflected by the expression and activity of antioxidant enzymes (SOD, GSH) and molecular anti-inflammatory pathways such as NF-*κ*B or Nrf-2 in the digestive system.

## 4. Future Perspectives

Due to its antioxidative and anti-inflammatory properties, CO released from its pharmacological donors or produced endogenously due to HO-1 activity seems to open new treatment modalities of GI tract disorders by exerting a strong protective potential which warrants its possible implementation in digestive system pharmacology. Detailed mechanism of CO-mediated gastroprotection against gastric mucosal I/R injury and possible DNA oxidation with special emphasis on the modulation of mitochondrial activity by this gas still remains unexplained. Moreover, novel ROS-sensitive CO prodrugs are promising tools for further investigation, perhaps in the treatment and prevention of various digestive system pathologies such as colitis, gastric mucosal injuries, postsurgical complications, and esophagitis [43]. Therefore, despite scientific evidence of its efficacy in the protection of mucosal components of digestive system, the detailed molecular mechanisms by which endogenous CO or CO-releasing donors exert antioxidative, gastroprotective, and/or therapeutic effects in the digestive system still require further studies. This could include interaction with nitric oxide or hydrogen sulfide.

## Figures and Tables

**Table 1:** Antioxidative effects of CO donors in various *in vitro* and *in vivo* experimental models of gastric mucosal injury or gastric cancer.

Experimental model (publication)	CO donor	Dose	Form of application
Ethanol-induced gastric damage mouse model [55]	DMDC	12.5 *μ*mol/kg	Intraperitoneal injection

↓ Lipid peroxidation, ↑ GSH			

Alendronate-induced gastric damage rat model [56]	DMDC	81 *μ*mol/kg	Intraperitoneal injection

↓ Lipid peroxidation, ↑ GSH			

Alendronate-induced gastric damage + mild stress rat model [30]	CORM-2	5 mg/kg	Intragastric injection

GPx-1 and SOD-2 gene expression not affected, ↓ NF-*κ*B gene expression			

Water immersion and restraint stress-induced gastric damage rat model [57]	CORM-2	1 mg/kg	Intragastric injection

**↓** Lipid peroxidation, restored activity of gastric mucosal SOD and GSH, and attenuated GPx-1 and SOD-2 gene expression			

Acute aspirin-induced gastric damage rat model [31]	CORM-2	5 mg/kg	Intragastric injection

↓ Lipid peroxidation, ↓ IL-1*β* gene expression, restored activity of GPx-1			

Human gastric adenocarcinoma (AGS) cell line [60]	CORM-2	10, 25, 50 *μ*M	Incubation with medium containing CO

↓ IL-1*β* induced IL-8 gene and protein expression, ↓ IL-8 promoter activity, ↓ IL-1*β* induced ROS production			

**Table 2:** Antioxidative effects of CO donors in various *in vitro* and *in vivo* experimental models of intestinal mucosa injury.

Experimental model (publication)	CO donor	Dose	Form of application
TNBS-induced colitis in mice [70]	CO gas	200 ppm	Inhalation

↓ Lipid peroxidation			

Cecal ligation and puncture-induced sepsis mouse model [68]	CORM-2	8 mg/kg	Intravenous injection

↓ Lipid peroxidation, ↓ IL-1*β* production			

Thermally induced small intestine injury mouse model [69]	CORM-2	8 mg/kg	Intravenous injection

↓Lipid peroxidation, ↓ IL-1*β* production, ↓ IL-8 production, and restored activity of GSH			

Cold I/R injury associated with small intestinal transplantation in rats [70]	CO gas	250 ppm	Inhalation

↑ Antioxidant power			

Hindlimb I/R-induced remote intestinal inflammatory response mouse model [71]	CO gas	250 ppm	Inhalation

No protection against intestinal lipid peroxidation			

Surgically induced postoperative ileus mouse model [73]	CORM-3	40 mg/kg	Intraperitoneal injection

↓ Lipid peroxidation			

TNF-*α*/cycloheximide-induced oxidative stress in the mouse small intestinal epithelial (MODE-K) cell line [74]	CORM-1A	100 *μ*M	Incubation with medium containing CO

↓ Intracellular ROS level, ↑ GSH			

**Table 3:** Antioxidative effects of CO donors in various *in vitro* and *in vivo* experimental models of liver injury.

Experimental model (publication)	CO donor	Dose	Form of application
Hindlimb I/R-induced systemic inflammation mouse model [85]	CO gas or MC	250 ppm or 5.8 *μ*mol/kg	Inhalation or orally

↓ Lipid peroxidation, ↓ inhibition of NADPH oxidase, and restored GSH/GSSG ratio			

Liver transplantation- (LTx-) induced I/R injury rat model/Kupffer cells isolated from the liver [86]	CO gas	20% CO-saturated culture medium	Incubation with medium containing CO

↓ ROS generation, ↑ HSP 70 protein expression			

Hemorrhagic shock and resuscitation rat model/Kupffer cells isolated from the liver [87]	CO gas	Gently bubbling CO gas through the RBC resuscitative fluid	Infusion of resuscitative fluids

↓ ROS generation			

Hepatic warm I/R injury mouse model [89]	CO gas	250 ppm	Inhalation

↑ ROS-dependent PI3 K/Akt activation, ↓ inhibition of GSK3*β* through Ser9 phosphorylation			

Ethanol-induced liver damage mouse model or primary rat hepatocytes [91]	CORM-2	8 mg/kg or 20 *μ*mol/l	Tail vein injection or incubation with medium containing CO

↓ Lipid peroxidation, restored GSH level, and restored SOD level			

APAP-induced liver injury in mice [79]	CORM-1A	20 mg/kg	Intraperitoneal injection

↑ Nrf2 gene upregulation, ↑ ARE gene upregulation, and restored GSH level			

HFHF diet-induced hepatic steatosis in mice [92]	CORM-1A	2 mg/kg/day	Intraperitoneal injection

↑ Nrf2 activation, ↑ ARE gene upregulation, and ↑ ATP production			

GalN/LPS-induced acute liver mouse model [94]	CO gas	First at a dose of 15 ml/kg, and then 6 h later, 8 ml/kg	Intraperitoneal injection

↓ Lipid peroxidation, restored GSH level, and restored SOD level			

Primary rat or mouse hepatocytes and Hep3B cells [96]	CO gas	250 ppm	Incubation with medium containing CO

↓ Apoptosis, ↓ endogenous antioxidant ascorbic acid, ↓ antioxidant power, ↑ ROS generation, ↑ Akt phosphorylation, and ↓ I*κ*B degradation (= ↑ NF-*κ*B activation)			

HepG2 cells [95]	CORM-2	80 *μ*M for 6 h	CO-saturated stock solutions

↑ Nrf2 activation, ↑ HO-1 expression			

Tert-butyl hydroperoxide- (t-BHP-) treated HepG2 cells [79]	CORM-A1	100 *μ*M	Incubation with medium containing CO

↓ ROS generation, ↑ Nrf2 activation			

Rat liver mitochondria [98]	CO gas	50 ppm for 1, 2, or 7 days	Inhalation

↓ GSH/GSSG ratio, ↑ activation of MMP, and ↑ mitochondrial SOD-2			

Mouse liver mitochondria [99]	CO solution	10 *μ*M	Swelling buffer

↓ Inhibition of MPP, ↑ mitochondrial ROS generation			

PA-treated HepG2 cells [92]	CORM-1A	100 *μ*M	Incubation with medium containing CO

↑ Nrf2 activation, ↑ ARE gene upregulation ↓ mitochondrial ROS generation, and ↑ activation of mitochondrial membrane potential			

**Table 4:** Antioxidative effects of CO donors in various *in vitro* and *in vivo* experimental models of pancreatic injury.

Experimental model (publication)	CO donor	Dose	Form of application
Retrograde infusion of sodium taurocholate-induced severe acute pancreatitis rat model [103]	CORM-2	8 mg/kg	Intravenous injection

↓ NF-*κ*B activity			

Retrograde injection of sodium taurocholate-induced acute necrotizing pancreatitis rat model [104]	MC	500 mg/kg	Orally

↓ NF-*κ*B activity			

Choline-deficient ethionine-supplemented diet-induced acute pancreatitis mouse model [105]	CO-HbV	1,000 mg Hb/kg	Via tail vein

↓ Oxidative stress			

## References

[1] Sies H. (2015). Oxidative stress: a concept in redox biology and medicine. *Redox Biology*.

[2] Sies H., Berndt C., Jones D. P. (2017). Oxidative stress. *Annual Review of Biochemistry*.

[3] Pizzino G., Irrera N., Cucinotta M. (2017). Oxidative stress: harms and benefits for human health. *Oxidative Medicine and Cellular Longevity*.

[4] Lushchak V. I. (2014). Free radicals, reactive oxygen species, oxidative stress and its classification. *Chemico-Biological Interactions*.

[5] Takaki A., Kawano S., Uchida D., Takahara M., Hiraoka S., Okada H. (2019). Paradoxical roles of oxidative stress response in the digestive system before and after carcinogenesis. *Cancers*.

[6] Liu J., Wang Z. (2015). Increased oxidative stress as a selective anticancer therapy. *Oxidative Medicine and Cellular Longevity*.

[7] Dikalov S. (2011). Cross talk between mitochondria and NADPH oxidases. *Free Radical Biology & Medicine*.

[8] Ighodaro O. M., Akinloye O. A. (2018). First line defence antioxidants-superoxide dismutase (SOD), catalase (CAT) and glutathione peroxidase (GPX): their fundamental role in the entire antioxidant defence grid. *Alexandria Journal of Medicine*.

[9] Sies H. (2017). Hydrogen peroxide as a central redox signaling molecule in physiological oxidative stress: oxidative eustress. *Redox Biology*.

[10] Wang Y., Branicky R., Noe A., Hekimi S. (2018). Superoxide dismutases: dual roles in controlling ROS damage and regulating ROS signaling. *The Journal of Cell Biology*.

[11] Kim Y. J., Kim E. H., Hahm K. B. (2012). Oxidative stress in inflammation-based gastrointestinal tract diseases: challenges and opportunities. *Journal of Gastroenterology and Hepatology*.

[12] Bhattacharyya A., Chattopadhyay R., Mitra S., Crowe S. E. (2014). Oxidative stress: an essential factor in the pathogenesis of gastrointestinal mucosal diseases. *Physiological Reviews*.

[13] Wu L., Wang R. (2005). Carbon monoxide: endogenous production, physiological functions, and pharmacological applications. *Pharmacological Reviews*.

[14] Dijkstra G., Blokzijl H., Bok L. (2004). Opposite effect of oxidative stress on inducible nitric oxide synthase and haem oxygenase-1 expression in intestinal inflammation: anti-inflammatory effect of carbon monoxide. *The Journal of Pathology*.

[15] Piantadosi C. A. (2008). Carbon monoxide, reactive oxygen signaling, and oxidative stress. *Free Radical Biology & Medicine*.

[16] Piantadosi C. A. (2002). Biological chemistry of carbon monoxide. *Antioxidants & Redox Signaling*.

[17] Pryor W. A., Houk K. N., Foote C. S. (2006). Free radical biology and medicine: it’s a gas, man!. *American Journal of Physiology. Regulatory, Integrative and Comparative Physiology*.

[18] Colpaert E. E., Timmermans J.-P., Lefebvre R. A. (2002). Immunohistochemical localization of the antioxidant enzymes biliverdin reductase and heme oxygenase-2 in human and pig gastric fundus. *Free Radical Biology and Medicine*.

[19] Hu Y., Yang M., Ma N., Shinohara H., Semba R. (1998). Contribution of carbon monoxide-producing cells in the gastric mucosa of rat and monkey. *Histochemistry and Cell Biology*.

[20] Takagi T., Naito Y., Mizushima K. (2008). Increased intestinal expression of heme oxygenase-1 and its localization in patients with ulcerative colitis. *Journal of Gastroenterology and Hepatology*.

[21] Bekyarova G., Tzaneva M., Hristova M. (2013). Heme oxygenase-1 expresion in gastric mucosa and liver after burns: preliminary immunohistochemical study. *Journal of Interdisciplinary Histopathology*.

[22] Rattan S., Fan Y. P., Chakder S. (1999). Mechanism of inhibition of VIP-induced LES relaxation by heme oxygenase inhibitor zinc protoporphyrin IX. *The American Journal of Physiology*.

[23] Fan Y. P., Chakder S., Rattan S. (1998). Inhibitory effect of zinc protoporphyrin IX on lower esophageal sphincter smooth muscle relaxation by vasoactive intestinal polypeptide and other receptor agonists. *The Journal of Pharmacology and Experimental Therapeutics*.

[24] Chang M., Xue J., Sharma V., Habtezion A. (2015). Protective role of hemeoxygenase-1 in gastrointestinal diseases. *Cellular and Molecular Life Sciences*.

[25] Wallace J. L., Ianaro A., de Nucci G. (2017). Gaseous mediators in gastrointestinal mucosal defense and injury. *Digestive Diseases and Sciences*.

[26] Motterlini R., Foresti R. (2017). Biological signaling by carbon monoxide and carbon monoxide-releasing molecules. *American Journal of Physiology. Cell Physiology*.

[27] Farrugia G., Szurszewski J. H. (2014). Carbon monoxide, hydrogen sulfide, and nitric oxide as signaling molecules in the gastrointestinal tract. *Gastroenterology*.

[28] Magierowska K., Brzozowski T., Magierowski M. (2018). Emerging role of carbon monoxide in regulation of cellular pathways and in the maintenance of gastric mucosal integrity. *Pharmacological Research*.

[29] Shen F., Zhao C. S., Shen M. F., Wang Z., Chen G. (2019). The role of hydrogen sulfide in gastric mucosal damage. *Medical Gas Research*.

[30] Magierowski M., Magierowska K., Szmyd J. (2016). Hydrogen sulfide and carbon monoxide protect gastric mucosa compromised by mild stress against alendronate injury. *Digestive Diseases and Sciences*.

[31] Magierowski M., Hubalewska-Mazgaj M., Magierowska K. (2018). Nitric oxide, afferent sensory nerves, and antioxidative enzymes in the mechanism of protection mediated by tricarbonyldichlororuthenium(II) dimer and sodium hydrosulfide against aspirin-induced gastric damage. *Journal of Gastroenterology*.

[32] Lanas A. (2008). Role of nitric oxide in the gastrointestinal tract. *Arthritis Research & Therapy*.

[33] Nemmani K. V. S., Mali S. V., Borhade N. (2009). NO-NSAIDs: gastric-sparing nitric oxide-releasable prodrugs of non-steroidal anti-inflammatory drugs. *Bioorganic & Medicinal Chemistry Letters*.

[34] Magierowski M., Magierowska K., Hubalewska-Mazgaj M. (2016). Interaction between endogenous carbon monoxide and hydrogen sulfide in the mechanism of gastroprotection against acute aspirin-induced gastric damage. *Pharmacological Research*.

[35] Magierowski M., Magierowska K., Hubalewska-Mazgaj M. (2018). Cross-talk between hydrogen sulfide and carbon monoxide in the mechanism of experimental gastric ulcers healing, regulation of gastric blood flow and accompanying inflammation. *Biochemical Pharmacology*.

[36] Magierowski M., Magierowska K., Hubalewska-Mazgaj M. (2017). Exogenous and endogenous hydrogen sulfide protects gastric mucosa against the formation and time-dependent development of ischemia/reperfusion-induced acute lesions progressing into deeper ulcerations. *Molecules*.

[37] Magierowska K., Korbut E., Hubalewska-Mazgaj M. (2019). Oxidative gastric mucosal damage induced by ischemia/reperfusion and the mechanisms of its prevention by carbon monoxide-releasing tricarbonyldichlororuthenium (II) dimer. *Free Radical Biology & Medicine*.

[38] Kobata A., Kotani T., Komatsu Y., Amagase K., Kato S., Takeuchi K. (2007). Dual action of nitric oxide in the pathogenesis of ischemia/reperfusion-induced mucosal injury in mouse stomach. *Digestion*.

[39] Magierowski M., Magierowska K., Surmiak M. (2017). The effect of hydrogen sulfide-releasing naproxen (ATB-346) versus naproxen on formation of stress-induced gastric lesions, the regulation of systemic inflammation, hypoxia and alterations in gastric microcirculation. *Journal of Physiology and Pharmacology*.

[40] Seo J. Y., Kim H., Seo J. T., Kim K. H. (2002). Oxidative stress induced cytokine production in isolated rat pancreatic acinar cells: effects of small-molecule antioxidants. *Pharmacology*.

[41] Motterlini R., Clark J. E., Foresti R., Sarathchandra P., Mann B. E., Green C. J. (2002). Carbon monoxide-releasing molecules. *Circulation Research*.

[42] Ji X., Pan Z., Li C. (2019). Esterase-sensitive and pH-controlled carbon monoxide prodrugs for treating systemic inflammation. *Journal of Medicinal Chemistry*.

[43] Pan Z., Zhang J., Ji K., Chittavong V., Ji X., Wang B. (2018). Organic CO prodrugs activated by endogenous ROS. *Organic Letters*.

[44] Babu D., Leclercq G., Motterlini R., Lefebvre R. A. (2017). Differential effects of CORM-2 and CORM-401 in murine intestinal epithelial MODE-K cells under oxidative stress. *Frontiers in Pharmacology*.

[45] Pan Z., Chittavong V., Li W. (2017). Organic CO prodrugs: structure-CO-release rate relationship studies. *Chemistry*.

[46] Ji X., Zhou C., Ji K. (2016). Click and release: a chemical strategy toward developing gasotransmitter prodrugs by using an intramolecular Diels-Alder reaction. *Angewandte Chemie International Edition*.

[47] Ji X., Ji K., Chittavong V., Yu B., Pan Z., Wang B. (2017). An esterase-activated click and release approach to metal-free CO-prodrugs. *Chemical Communications*.

[48] Orlando R. C. (2010). The integrity of the esophageal mucosa. Balance between offensive and defensive mechanisms. *Best Practice & Research. Clinical Gastroenterology*.

[49] Yoshida N. (2007). Inflammation and oxidative stress in gastroesophageal reflux disease. *Journal of Clinical Biochemistry and Nutrition*.

[50] Liu G., Jiang C., Li D. (2019). Isorhamnetin alleviates esophageal mucosal injury in a chronic model of reflux esophagitis. *European Journal of Pharmacology*.

[51] Song H. J., Shin C. Y., Oh T. Y., Min Y. S., Park E. S., Sohn U. D. (2009). Eupatilin with heme oxygenase-1-inducing ability protects cultured feline esophageal epithelial cells from cell damage caused by indomethacin. *Biological & Pharmaceutical Bulletin*.

[52] Hunt R. H., Camilleri M., Crowe S. E. (2015). The stomach in health and disease. *Gut*.

[53] Suzuki H., Nishizawa T., Tsugawa H., Mogami S., Hibi T. (2012). Roles of oxidative stress in stomach disorders. *Journal of Clinical Biochemistry and Nutrition*.

[54] Ueda K., Ueyama T., Yoshida K. (2008). Adaptive HNE-Nrf2-HO-1 pathway against oxidative stress is associated with acute gastric mucosal lesions. *American Journal of Physiology. Gastrointestinal and Liver Physiology*.

[55] Gomes A. S., Gadelha G. G., Lima S. J. (2010). Gastroprotective effect of heme-oxygenase 1/biliverdin/CO pathway in ethanol-induced gastric damage in mice. *European Journal of Pharmacology*.

[56] Costa N. R. D., Silva R. O., Nicolau L. A. D. (2013). Role of soluble guanylate cyclase activation in the gastroprotective effect of the HO-1/CO pathway against alendronate-induced gastric damage in rats. *European Journal of Pharmacology*.

[57] Kwiecien S., Magierowska K., Magierowski M. (2016). Role of sensory afferent nerves, lipid peroxidation and antioxidative enzymes in the carbon monoxide-induced gastroprotection against stress ulcerogenesis. *Journal of Physiology and Pharmacology*.

[58] Hwang Y. S., Jeong M., Park J. S. (2004). Interleukin-1beta stimulates IL-8 expression through MAP kinase and ROS signaling in human gastric carcinoma cells. *Oncogene*.

[59] Zanellato I., Bonarrigo I., Ravera M., Gabano E., Gust R., Osella D. (2013). The hexacarbonyldicobalt derivative of aspirin acts as a CO-releasing NSAID on malignant mesothelioma cells. *Metallomics*.

[60] Lian S., Xia Y., Ung T. T. (2016). Carbon monoxide releasing molecule-2 ameliorates IL-1*β*-induced IL-8 in human gastric cancer cells. *Toxicology*.

[61] Gasbarrini G., Montalto M., Santoro L. (2008). Intestine: organ or apparatus?. *Digestive Diseases*.

[62] Gibbons S. J., Verhulst P. J., Bharucha A., Farrugia G. (2013). Review article: carbon monoxide in gastrointestinal physiology and its potential in therapeutics. *Alimentary Pharmacology & Therapeutics*.

[63] Naito Y., Takagi T., Yoshikawa T. (2004). Heme oxygenase-1: a new therapeutic target for inflammatory bowel disease. *Alimentary Pharmacology and Therapeutics*.

[64] Westbrook A. M., Szakmary A., Schiestl R. H. (2010). Mechanisms of intestinal inflammation and development of associated cancers: lessons learned from mouse models. *Mutation Research/Reviews in Mutation Research*.

[65] Hegazi R. A., Rao K. N., Mayle A., Sepulveda A. R., Otterbein L. E., Plevy S. E. (2005). Carbon monoxide ameliorates chronic murine colitis through a heme oxygenase 1-dependent pathway. *The Journal of Experimental Medicine*.

[66] Takagi T., Naito Y., Mizushima K. (2010). Inhalation of carbon monoxide ameliorates TNBS-induced colitis in mice through the inhibition of TNF-*α* expression. *Digestive Diseases and Sciences*.

[67] Yin H., Fang J., Liao L., Nakamura H., Maeda H. (2014). Styrene-maleic acid copolymer-encapsulated CORM2, a water-soluble carbon monoxide (CO) donor with a constant CO-releasing property, exhibits therapeutic potential for inflammatory bowel disease. *Journal of Controlled Release*.

[68] Wang X., Cao J., Sun B. W., Liu D. D., Liang F., Gao L. (2012). Exogenous carbon monoxide attenuates inflammatory responses in the small intestine of septic mice. *World Journal of Gastroenterology*.

[69] Liu D. M., Sun B. W., Sun Z. W., Jin Q., Sun Y., Chen X. (2008). Suppression of inflammatory cytokine production and oxidative stress by CO-releasing molecules-liberated CO in the small intestine of thermally-injured mice. *Acta Pharmacologica Sinica*.

[70] Nakao A., Kimizuka K., Stolz D. B. (2003). Carbon monoxide inhalation protects rat intestinal grafts from ischemia/reperfusion injury. *The American Journal of Pathology*.

[71] Scott J. R., Cukiernik M. A., Ott M. C. (2009). Low-dose inhaled carbon monoxide attenuates the remote intestinal inflammatory response elicited by hindlimb ischemia-reperfusion. *American Journal of Physiology. Gastrointestinal and Liver Physiology*.

[72] van Bree S. H., Nemethova A., Cailotto C., Gomez-Pinilla P. J., Matteoli G., Boeckxstaens G. E. (2012). New therapeutic strategies for postoperative ileus. *Nature Reviews. Gastroenterology & Hepatology*.

[73] De Backer O., Elinck E., Blanckaert B., Leybaert L., Motterlini R., Lefebvre R. A. (2009). Water-soluble CO-releasing molecules reduce the development of postoperative ileus via modulation of MAPK/HO-1 signalling and reduction of oxidative stress. *Gut*.

[74] Babu D., Soenen S. J., Raemdonck K. (2012). TNF-*α*/cycloheximide-induced oxidative stress and apoptosis in murine intestinal epithelial MODE-K cells. *Current Pharmaceutical Design*.

[75] Babu D., Motterlini R., Lefebvre R. A. (2015). CO and CO-releasing molecules (CO-RMs) in acute gastrointestinal inflammation. *British Journal of Pharmacology*.

[76] Babu D., Leclercq G., Goossens V. (2015). Antioxidant potential of CORM-A1 and resveratrol during TNF-*α*/cycloheximide-induced oxidative stress and apoptosis in murine intestinal epithelial MODE-K cells. *Toxicology and Applied Pharmacology*.

[77] Liu H., Liu X., Zhang C. (2017). Redox imbalance in the development of colorectal cancer. *Journal of Cancer*.

[78] Yan F., Zhang Q. Y., Jiao L. (2009). Synergistic hepatoprotective effect of _Schisandrae_ lignans with *Astragalus* polysaccharides on chronic liver injury in rats. *Phytomedicine*.

[79] Upadhyay K. K., Jadeja R. N., Thadani J. M. (2018). Carbon monoxide releasing molecule A-1 attenuates acetaminophen-mediated hepatotoxicity and improves survival of mice by induction of Nrf2 and related genes. *Toxicology and Applied Pharmacology*.

[80] Singh D., Cho W. C., Upadhyay G. (2016). Drug-induced liver toxicity and prevention by herbal antioxidants: an overview. *Frontiers in Physiology*.

[81] Farzaei M., Zobeiri M., Parvizi F. (2018). Curcumin in liver diseases: a systematic review of the cellular mechanisms of oxidative stress and clinical perspective. *Nutrients*.

[82] Eltzschig H. K., Eckle T. (2011). Ischemia and reperfusion—from mechanism to translation. *Nature Medicine*.

[83] Jaeschke H., Woolbright B. L. (2012). Current strategies to minimize hepatic ischemia-reperfusion injury by targeting reactive oxygen species. *Transplantation Reviews*.

[84] Bauer M., Bauer I. (2002). Heme oxygenase-1: redox regulation and role in the hepatic response to oxidative stress. *Antioxidants & Redox Signaling*.

[85] Brugger J., Schick M. A., Brock R. W. (2010). Carbon monoxide has antioxidative properties in the liver involving p38 MAP kinase pathway in a murine model of systemic inflammation. *Microcirculation*.

[86] Lee L. Y., Kaizu T., Toyokawa H. (2011). Carbon monoxide induces hypothermia tolerance in Kupffer cells and attenuates liver ischemia/reperfusion injury in rats. *Liver Transplantation*.

[87] Ogaki S., Taguchi K., Maeda H. (2015). Kupffer cell inactivation by carbon monoxide bound to red blood cells preserves hepatic cytochrome P450 *via* anti-oxidant and anti-inflammatory effects exerted through the HMGB1/TLR-4 pathway during resuscitation from hemorrhagic shock. *Biochemical Pharmacology*.

[88] Kato Y., Shimazu M., Kondo M. (2003). Bilirubin rinse: a simple protectant against the rat liver graft injury mimicking heme oxygenase-1 preconditioning. *Hepatology*.

[89] Kim H. J., Joe Y., Kong J. S. (2013). Carbon Monoxide Protects against Hepatic Ischemia/Reperfusion Injury via ROS- Dependent Akt Signaling and Inhibition of Glycogen Synthase Kinase 3*β*. *Oxidative Medicine and Cellular Longevity*.

[90] Kim H. J., Joe Y., Yu J. K. (2015). Carbon monoxide protects against hepatic ischemia/reperfusion injury by modulating the miR-34a/SIRT1 pathway. *Biochimica et Biophysica Acta*.

[91] Li Y., Gao C., Shi Y. (2013). Carbon monoxide alleviates ethanol-induced oxidative damage and inflammatory stress through activating p38 MAPK pathway. *Toxicology and Applied Pharmacology*.

[92] Upadhyay K. K., Jadeja R. N., Vyas H. S. (2020). Carbon monoxide releasing molecule-A1 improves nonalcoholic steatohepatitis via Nrf2 activation mediated improvement in oxidative stress and mitochondrial function. *Redox Biology*.

[93] Sass G., Seyfried S., Parreira Soares M. (2004). Cooperative effect of biliverdin and carbon monoxide on survival of mice in immune-mediated liver injury. *Hepatology*.

[94] Wen Z., Liu Y., Li F., Wen T. (2013). Low dose of carbon monoxide intraperitoneal injection provides potent protection against GalN/LPS-induced acute liver injury in mice. *Journal of Applied Toxicology*.

[95] Lee B. S., Heo J., Kim Y. M. (2006). Carbon monoxide mediates heme oxygenase 1 induction via Nrf2 activation in hepatoma cells. *Biochemical and Biophysical Research Communications*.

[96] Kim H. S., Loughran P. A., Rao J., Billiar T. R., Zuckerbraun B. S. (2008). Carbon monoxide activates NF-*κ*B via ROS generation and Akt pathways to protect against cell death of hepatocytes. *American Journal of Physiology. Gastrointestinal and Liver Physiology*.

[97] Bellezza I., Mierla A. L., Minelli A. (2010). Nrf2 and NF-*κ*B and their concerted modulation in Cancer pathogenesis and progression. *Cancers*.

[98] Piantadosi C. A., Carraway M. S., Suliman H. B. (2006). Carbon monoxide, oxidative stress, and mitochondrial permeability pore transition. *Free Radical Biology & Medicine*.

[99] Queiroga C. S. F., Almeida A. S., Alves P. M., Brenner C., Vieira H. L. A. (2011). Carbon monoxide prevents hepatic mitochondrial membrane permeabilization. *BMC Cell Biology*.

[100] Bastidas-Ponce A., Scheibner K., Lickert H., Bakhti M. (2017). Cellular and molecular mechanisms coordinating pancreas development. *Development*.

[101] Pereda J., Sabater L., Aparisi L. (2006). Interaction between cytokines and oxidative stress in acute pancreatitis. *Current Medicinal Chemistry*.

[102] Sato H., Siow R. C., Bartlett S. (1997). Expression of stress proteins heme oxygenase-1 and -2 in acute pancreatitis and pancreatic islet betaTC3 and acinar AR42J cells. *FEBS Letters*.

[103] Chen P., Sun B., Chen H. (2010). Effects of carbon monoxide releasing molecule-liberated CO on severe acute pancreatitis in rats. *Cytokine*.

[104] Nuhn P., Mitkus T., Ceyhan G. O. (2013). Heme oxygenase 1–generated carbon monoxide and biliverdin attenuate the course of experimental necrotizing pancreatitis. *Pancreas*.

[105] Nagao S., Taguchi K., Sakai H. (2016). Carbon monoxide-bound hemoglobin vesicles ameliorate multiorgan injuries induced by severe acute pancreatitis in mice by their anti-inflammatory and antioxidant properties. *International Journal of Nanomedicine*.

[106] Nikolic I., Saksida T., Mangano K. (2014). Pharmacological application of carbon monoxide ameliorates islet-directed autoimmunity in mice via anti-inflammatory and anti-apoptotic effects. *Diabetologia*.

[107] Li M., Peterson S., Husney D. (2007). Interdiction of the diabetic state in NOD mice by sustained induction of heme oxygenase: possible role of carbon monoxide and bilirubin. *Antioxidants & Redox Signaling*.

